# Supplementary material for: Causal Relationship between Aging and Anorexia Nervosa: A White-Matter-Microstructure-Mediated Mendelian Randomization Analysis
Source: Biomedicines. 2024 Aug 16;12(8):1874. doi: 10.3390/biomedicines12081874 (PMC11351342; doi:10.3390/biomedicines12081874)
Supplement: Supplementary file 1 [file biomedicines-12-01874-s001.zip › Supplementary Figure/Figure S4/Singe SNP plot of MR analysis for TL to ubm-b-1660.pdf]

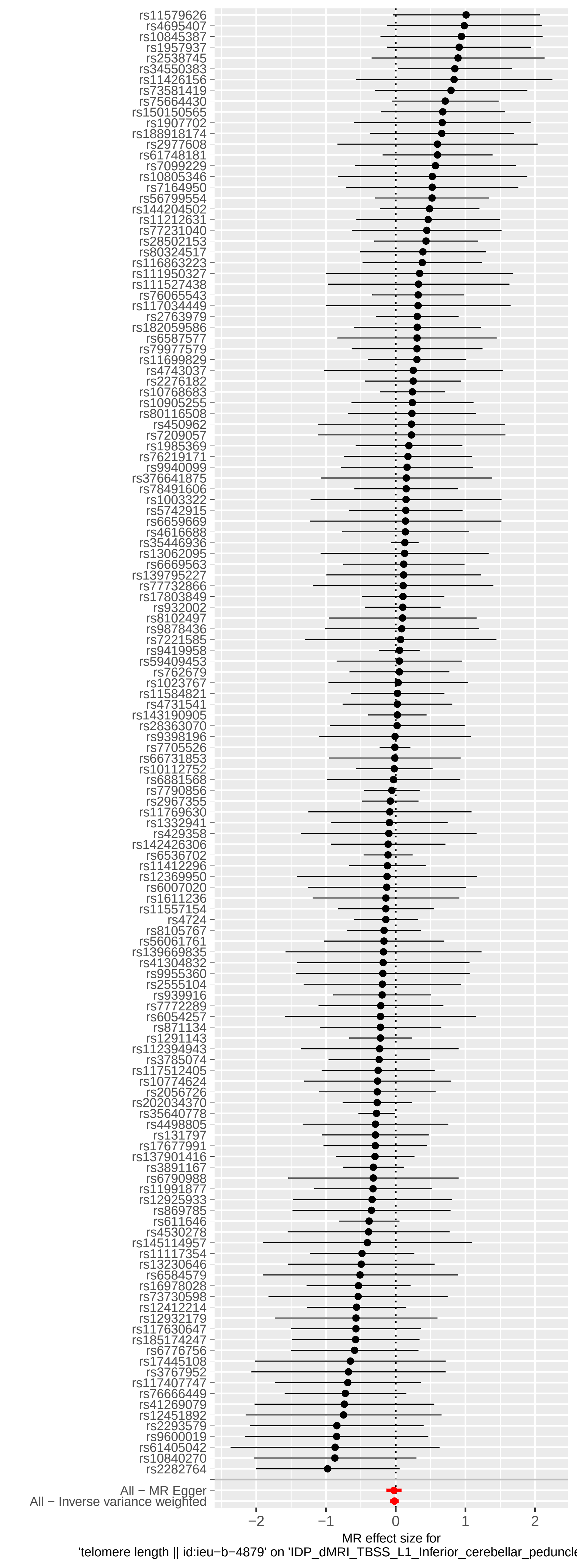
rs11579626
rs4695407
rs10845387
rs1957937
rs2538745
rs34550383
rs11426156
rs73581419
rs75664430
rs150150565
rs1907702
rs188918174
rs2977608
rs61748181
rs7099229
rs10805346
rs7164950
rs56799554
rs144204502
rs11212631
rs77231040
rs28502153
rs80324517
rs116863223
rs111950327
rs111527438
rs76065543
rs117034449
rs2763979
rs182059586
rs6587577
rs79977579
rs11699829
rs4743037
rs2276182
rs10768683
rs10905255
rs80116508
rs450962
rs7209057
rs1985369
rs76219171
rs9940099
rs376641875
rs78491606
rs1003322
rs5742915
rs6659669
rs4616688
rs35446936
rs13062095
rs6669563
rs139795227
rs77732866
rs17803849
rs932002
rs8102497
rs9878436
rs7221585
rs9419958
rs59409453
rs762679
rs1023767
rs11584821
rs4731541
rs143190905
rs28363070
rs9398196
rs7705526
rs66731853
rs10112752
rs6881568
rs7790856
rs2967355
rs11769630
rs1332941
rs429358
rs142426306
rs6536702
rs11412296
rs12369950
rs6007020
rs1611236
rs11557154
rs4724
rs8105767
rs56061761
rs139669835
rs41304832
rs9955360
rs2555104
rs939916
rs7772289
rs6054257
rs871134
rs1291143
rs112394943
rs3785074
rs117512405
rs10774624
rs2056726
rs202034370
rs35640778
rs4498805
rs131797
rs17677991
rs137901416
rs3891167
rs6790988
rs11991877
rs12925933
rs869785
rs611646
rs4530278
rs145114957
rs11117354
rs13230646
rs6584579
rs16978028
rs73730598
rs12412214
rs12932179
rs117630647
rs185174247
rs6776756
rs17445108
rs3767952
rs117407747
rs76666449
rs41269079
rs12451892
rs2293579
rs9600019
rs61405042
rs10840270
rs2282764
All − MR Egger
All − Inverse variance weighted
−2
−1
0
1
2
MR effect size for
'telomere length || id:ieu−b−4879' on 'IDP_dMRI_TBSS_L1_Inferior_cerebellar_peduncle
